# Antimicrobial Use and Antimicrobial Resistance Indicators—Integration of Farm-Level Surveillance Data From Broiler Chickens and Turkeys in British Columbia, Canada

**DOI:** 10.3389/fvets.2019.00131

**Published:** 2019-05-03

**Authors:** Agnes Agunos, Sheryl P. Gow, David F. Léger, Carolee A. Carson, Anne E. Deckert, Angelina L. Bosman, Daleen Loest, Rebecca J. Irwin, Richard J. Reid-Smith

**Affiliations:** Public Health Agency of Canada, Center for Foodborne, Environmental and Zoonotic Infectious Diseases, Guelph, ON, Canada

**Keywords:** metrics, indicators, farm-level, surveillance, Canada

## Abstract

Using data from the Canadian Integrated Program for Antimicrobial Resistance Surveillance (CIPARS), we aimed to describe trends in antimicrobial use (AMU) in broiler chickens and turkeys, to compare AMU across species, to compare with trends in antimicrobial resistance (AMR), and to assess the effects of various AMU/AMR units of measurement (metrics and indicators) on data integration. Data on AMU and AMR in enteric bacteria, collected from 2013 to 2017 from broiler chickens (*n* = 143 flocks) and turkeys (*n* = 145) were used. In broiler chickens, the total AMU in milligrams/population correction unit (mg/PCU_Br_) decreased by 6%, the number (*n*) of defined daily doses for animals using Canadian standards (nDDDvetCA) per 1,000 broiler chicken-days decreased by 12%, and nDDDvetCA/PCU decreased by 6%. In turkeys, the mg/PCU_Tk_ decreased by 1%, whereas the nDDDvetCA/1,000 turkey-days and the nDDDvetCA/PCU increased by 1 and 5%, respectively. The types of antimicrobial classes used in both species were similar. Using the frequency of flocks reporting use (i.e., number of flocks reporting use/number of flocks participating) as a measurement, the use of certain antimicrobials changed over time (e.g., Broilers, decreased cephalosporin use, virginiamycin use, emerging use of lincomycin-spectinomycin, and avilamycin; Turkeys: increased trimethoprim-sulfonamides and macrolide use). The trends in resistance to specific antimicrobials paralleled the frequency and quantity of use (e.g., ceftriaxone use decreased—ceftriaxone resistance decreased, and gentamicin use increased—gentamicin resistance increased) in some situations, but not others (decreased fluoroquinolone use—increased ciprofloxacin resistance). AMR data were summarized using the AMR indicator index (AMR Ix). The most notable AMR Ix trend was the decrease in ceftriaxone AMR Ix among *Escherichia coli* (0.19 to 0.07); indicative of the success of the poultry industry action to eliminate the preventive use of third generation cephalosporins. Other trends observed were the increase in ciprofloxacin AMR Ix among *Campylobacte*r from 0.23 to 0.41 and gentamicin AMR Ix among *E. coli* from 0.11 to 0.22, suggestive of the persistence/emergence of resistance related to previous and current AMU not captured in our surveillance timeframe. These data highlight the necessity of multiple AMU and AMR indicators for monitoring the impact of stewardship activities and interventions.

## Introduction

Strengthening current surveillance capacities and expertise in antimicrobial use (AMU) and antimicrobial resistance (AMR) is one of the strategic objectives identified in “Tackling Antimicrobial Resistance and Antimicrobial Use: A Pan-Canadian Framework for Action” (1). This effort aligns with the global call to address AMR, such as the World Health Organization's (WHO) Global Action Plan (GAP) on AMR (2), the Food and Agriculture Organization of the United Nation's (FAO) action plan on AMR (3), and the World Organisation for Animal Health's (OIE) strategy on AMR and the prudent use of antimicrobials (4). The tripartite alliance (FAO-OIE-WHO), in the context of “One Health,” is jointly addressing emerging threats in the animal-environment-human interface and identified AMR as one of the initial priority areas for collaboration (5). Canada's Framework for Action involves multi-stakeholder engagement and collaboration (both government and industry), to collectively address AMR.

Many countries have established surveillance systems for AMR in food animals (6–9). Similarly, for AMU surveillance, there are many activities at the global, regional, and national levels involving data collection, reporting and development of AMU metrics and indicators. In 2017, the OIE published its 2nd annual report on the use of antimicrobial agents (10), wherein the global data on the quantity of antimicrobials used in animals weighted by biomass, and stratified by region, were reported for the first time (10). In Europe, the European Medicines Agency (EMA)'s European Surveillance for Veterinary Antimicrobial Consumption (ESVAC project) provides guidance for AMU monitoring (11), and AMU data collection and reporting (12). As suggested in the revised ESVAC reflection paper, AMU data should be collected at the farm level to assess temporal trends and understand overall AMU context and impacts of interventions in terms of prudent use/stewardship (13). Collaborative efforts to address AMR, such as the Joint Programming Initiative on Antimicrobial Resistance (JPI-AMR) contribute to the implementation of WHO's GAP (14). One project relevant to AMU surveillance in animals arising from JPI-AMR is the AACTING project (network on quantification, benchmarking, and reporting of veterinary AMU at farm level), which developed a guideline document on AMU data collection and measurements at the farm level (15).

Once national action plans (NAP) have been developed and implemented (16), it is expected that surveillance systems will be progressively strengthened and that comprehensive data (metadata) will be generated, enabling data integration from surveillance programs across sectors to monitor the overall progress of national or regional interventions to address AMR. In June 2017, the European Centre for Disease Control (ECDC), European Food Safety Authority (EFSA), and EMA published their 2nd Joint Interagency Antimicrobial Consumption and Resistance Analysis (JIACRA Report), integrating AMU and AMR data across animal species and in humans (17), followed by the “Joint Scientific Opinion on a list of outcome indicators as regards surveillance of antimicrobial resistance and antimicrobial consumption in humans and food producing animals” (18).

For AMU surveillance, metrics (the technical units of measurement, such as frequency of use) and indicators (an AMU metric in relation to a denominator, such as animal biomass or animal time unit described below) have been developed. Milligrams weighted by population and weight (mg/PCU) is used for reporting national sales and distribution data across countries in the European Union (11). Another AMU indicator is treatment incidence (TI), which pertains to the total number of defined daily doses in animals adjusted for animal-time units (19–21). The number of defined daily doses in animals per PCU is an AMU measurement to monitor AMU sales data in animals (17). Requirements for AMU measurements vary depending on surveillance objectives and include spatial and temporal resolution (frequency on which AMU data are collected), comprehensiveness (capacity to collect usage data from all units in the target population), stability over time, and comparability between populations (22). An AMR indicator is a summarized AMR measurement integrating select AMR data across different bacterial species (e.g., of public health importance) at the national level, aimed at monitoring national and multi-stakeholder stewardship efforts and initiatives to mitigate AMR risks (17). The antimicrobial resistance indicator index (AMR Ix) is a novel AMR indicator, calculated as the percentage of resistance (or susceptibility) to a certain antimicrobial/s, adjusted by PCU (18).

In Canada, CIPARS (Canadian Integrated Program for Antimicrobial Resistance Surveillance) collects, analyses, and communicates trends in AMU and AMR for select bacteria from humans, and food animals along the production continuum (23). The broiler chicken farm component of CIPARS was initiated in 2013 in the major poultry producing provinces in Canada, including British Columbia (BC). In addition to broiler chickens, samples were also collected from the turkey sector of the poultry industry in BC. The farm component was initiated prior to the May 2014 implementation of the first step of the poultry industry AMU strategy, which entailed eliminating the preventive use of Health Canada's Veterinary Drugs Directorate (VDD) Category I antimicrobials (e.g., 3rd generation cephalosporins and fluoroquinolones) (24, 25). Veterinary antimicrobials used in Canada are categorized by Health Canada's Veterinary Drugs Directorate (VDD) according to their importance to human medicine (VDD Category I-very high importance, Category II-high importance, Category III-medium importance, and VDD Category IV—low importance) (26). The second and third steps of the poultry industry AMU strategy aim to eliminate the preventive use of VDD Category II antimicrobials (e.g., aminoglycosides, streptogramins, macrolides, penicillins, trimethoprim-sulfonamides) by the end of 2018 (broiler chickens and turkeys), and Category III antimicrobials (e.g., bacitracins, tetracyclines, sulfonamides) by the end of 2019 for turkeys and 2020 for broiler chickens (i.e., contingent upon reassessment of this preventive strategy on production metrics and AMR prevalence by the end of 2019 in broiler chickens) (25). The data generated during 2013–2017 enabled analyses of various AMU and AMR metrics and indicators to measure the impact of the initial intervention step by the poultry industry. The objective of this study was to describe AMU trends (2013–2017) in poultry sampled through CIPARS in BC, compare AMU between poultry species, describe AMR over time, and to compare potential AMU and AMR indicators for data integration. This work will inform the selection of AMU and AMR indicators to best monitor the progress of the implementation of industry (24, 25) and government initiatives (e.g., enhanced veterinary oversight, prescription of antimicrobials belonging to VDD Categories I to III) to address AMR (27, 28), and will serve as a reference point in BC to measure the future impacts of the poultry industry's on-going AMU reduction strategy (24, 25).

## Materials and Methods

Poultry data used in the analysis were collected through CIPARS from BC between 2013 and 2017. From this point forward, poultry refers to combined data from commercial broiler chickens and turkeys, unless indicated otherwise.

### Farm and Flock Selection

Prior to farm enrollment, veterinarians participating in the CIPARS farm program administered an informed consent to the producers. Briefly, each year, 30 broiler flocks and 30 turkey flocks in BC were selected for surveillance. This is proportional to the broiler and turkey production profiles of the province (29, 30) compared to the rest of Canada, based on a sampling framework described elsewhere (9, 31). One flock per farm was visited by the veterinarian each year. The participating CIPARS veterinarians (*n* = 4) represented 100% of the poultry veterinary practices in BC. A flock, assigned with a unique code (i.e., identity is known only to the veterinarian), is defined as a group of broiler or turkey birds, hatched and placed in the designated production unit (e.g., floor, pen, barn) approximately the same day. A farm is a registered establishment that may have one or more barns in the premise. For farm selection, veterinarians were instructed to follow certain inclusion and exclusion criteria. The farms must be a commercial quota-holding operation (backyard and small flocks excluded) and compliant to on-farm food safety program (e.g., Safe, Safer, Safest™, the Chicken Farmers of Canada's on-farm food safety assurance program and Turkey Farmers of Canada's On Farm Food Safety Program^©^) (32, 33). Various production systems (antibiotic-free [ABF], raised without antibiotics [RWA] or organic production) were included but veterinarians were instructed to select the number of flocks proportional to their practice profile. Veterinarians ensured that selected farms were representative of all the Canadian Hatcheries Federation member hatcheries supplying chicks and *poults* and representative of the feed mills supplying feeds in BC, and were geographically distributed across the province (i.e., farms selected do not cluster in one administrative district). The final criteria ensured that farms selected were demographically reflective of the veterinary practice and varied in terms of flock and farm capacity, animal health programs, biosecurity measures, management practices, and production efficiency parameters (e.g., poorly managed to best managed flocks). As previously described (9, 31), these criteria helped ensure that the flocks enrolled were representative of most broiler chicken and turkey flocks raised in BC. Veterinarians were also instructed to distribute their sampling visits across the year to account for seasonal variations of disease pressures that may drive AMU.

### Farm Surveillance Design and Laboratory Methods

Antimicrobial use, pathogen recovery and AMR data were obtained from the same flocks. A species-specific farm questionnaire (9, 31) was used to collect farm AMU and relevant production, animal health and biosecurity information. Flocks were sampled at least 1 week prior to shipment; this stage of production is closest to the consumer and also ensures that AMU until the end or last stages of the production period is captured in the questionnaire. In turkeys, all Turkey Farmers of Canada's marketing weight categories (30) were included in the sampling framework (e.g., broiler turkeys, light hens, heavy hens, light toms, heavy toms). At the time of the farm visit, pooled fecal samples were collected according to routine CIPARS farm protocol described elsewhere (9, 23, 31). In brief, 4 pooled fresh fecal samples representing the 4 quadrant of the barn were collected per flock. Each sample was cultured for *Escherichia coli, Salmonella*, and *Campylobacter*. Antimicrobial susceptibility testing was conducted using Minimum inhibitory concentrations (MIC) using an automated broth microdilution and the Clinical and Laboratory Standard Institute (CLSI) M7-A8 standards and breakpoints when available (9, 23, 31). Susceptibility of *E. coli* and *Salmonella* isolates was tested using the CMV3AGNF plate (contained 14 antimicrobials) and susceptibility of *Campylobacter* isolates was tested using the NARMS CAMPY plates (contained 9 antimicrobials) (Sensititre; Trek Diagnostic Systems, West Sussex, England) designed by the National Antimicrobial Resistance Monitoring System (NARMS) of the United States (9, 23).

### Data Sources

#### AMU Data

Information on AMU for broiler chickens and turkeys were extracted from the CIPARS farm surveillance PostGreSQL database designed to capture the questionnaire survey data into Microsoft Excel (Office 14). The characteristics of the data collected, and detailed data collection methods are described elsewhere (9, 23). For the AMU data used in this study, count data (farms, rations, days treated), and quantitative data (inclusion rates, milligrams of antimicrobial active ingredient and class) were extracted from the database.

#### AMR Data

Bacterial isolation and AMR information from flock samples were extracted from the Public Health Agency of Canada's data repository (Data Extraction and Analysis System).

### Data Analysis

#### AMU Metrics and Indicators

AMU metrics utilized in this document were count-based (i.e., frequency of flocks), and the weight- or dose-based AMU indicators described in Equations 1–4. For the current paper, the Category IV antimicrobials (e.g., ionophores) and antimicrobials with no classification at the time of writing of this report (e.g., chemical coccidiostats, arsenicals, and pyrimethamine) were excluded.

**Frequency of use (number of flocks reporting AMU/total number of flocks sampled):** This count-based AMU metric was created for each poultry species and stratified by antimicrobial and route of administration.

**Weight-Based Indicator**. The mg/PCU was used to compare trends in AMU quantity between broiler chickens (mg/PCU_Br_) and turkeys (mg/PCU_Tk_) and the total poultry AMU (mg/PCU_poultry_) in BC.

**mg/PCU (by species):** This was derived by dividing the total milligrams (mg) of antimicrobial active ingredient (AAI) administered by the biomass or PCU as per the ESVAC methodology for calculating national sales and distribution data (11). As per routine CIPARS analysis (9, 31), the PCU is calculated as the total population (minus half the cumulative mortalities recorded at the time of the farm visit) multiplied by 1 kg or 6.5 kg ESVAC standard weight at treatment for broiler chickens and turkeys, respectively. These species-specific denominators or “species PCU” described in the ESVAC's “Guidance on collection and provision of national data on antimicrobial use by animal species/categories” (12), was used to estimate the AMU quantity in broiler chickens (mg/PCU_Br_) and turkeys (mg/PCU_Tk_). This measure was also estimated per antimicrobial class, and for specific antimicrobials, such as TIO, GEN, and LINC-SPEC.

**Equation 1. milligrams/population correction unit by species** (mg/PCU_Br_, mg/PCU_Tk_)

$$\begin{array}{l} mg/PCU=\;\frac{AAI\;in\;feed\;\left(mg\right)\;+\;water\;\left(mg\right)+injection\;\left(mg\right)\;}{PCU\left(Total\;population\;\times standard\;weight\;in\;kg\right)} \end{array}$$

**mg/PCU**_**poultry**_**:** sum of the amount of AAI (mg) administered to broiler chickens and turkeys divided by the total poultry biomass.

**Equation 2. mg/PCU**_**Poultry**_

$$\begin{array}{l} mg/PCU_{poultry}=\;\frac{\sum mg\;AAI\;administered\;to\;broiler\;chickens\;and\;turkeys\;}{\sum PCU\;of\;broiler\;chickens\;and\;turkeys}\; \end{array}$$

**Dose-Based Indicators**. Two dose-based indicators, nDDDvetCA/1,000 animal-days at risk and nDDDvetCA/PCU, were calculated to assess trends over time and comparability of the AMU data in broiler chickens and turkeys.

**nDDDvetCA/1,000 animal-days at risk** (nDDDvetCA/1,000 broiler chicken-days at risk and nDDDvetCA/1,000 turkey-days at risk)**:** This dose-based indicator was calculated by dividing the DDDvetCA (mg/kg/day) by the biomass and time-animal unit (specific days at risk for each species; this is equivalent to the age in days at pre-harvest sampling). As previously described (31), each antimicrobial was assigned a DDDvetCA following similar methodology to ESVAC's DDDvet assignment, by obtaining the average of all approved unique doses (for prevention and treatment purposes) based on Canadian drug product inserts (34, 35). The nDDDvetCA was calculated by dividing the amount of AAI used (mg) by the DDDvetCA (mg/kg/day). The DDDvetCA standards are listed in the Supplementary Materials, Annex 1.

**Equation 3. nDDDvetCA/1,000 animal-days at risk by species** (nDDDvetCA/1,000 broiler chicken-days at risk and nDDDvetCA/1,000 turkey-days at risk)

$$\begin{array}{l} {nDDDvetCA/}_{1,000\;animal-days\;at\;risk}\\ =\left(\frac{{total\;antimicrobials\;\left(mg\right)/DDDvetCA}_{mg/kg/day}}{total\;animals\;\times ESVAC\;std.\;weight\;\left(kg\right)\times days\;at\;risk}\right)\times 1,000\; \end{array}$$

The average broiler chicken-days at risk used in the above calculations were 33–34 days depending on the year (as reported in the surveillance data). Average turkey-days at risk used in the above calculations were 84–89 days depending on the year (as reported in the surveillance data).

**nDDDvetCA/PCU:** This dose-based indicator was derived from the amount of AAI used (mg) divided by the DDDvetCA standard and the animal biomass. This was calculated for each species.

**Equation 4. nDDDvetCA/PCU by species** (nDDDvetCA/PCU_Br_ and nDDDvetCA/PCU_Tk_)

$$\begin{array}{l} nDDDvetCA/PCU=\frac{\left(Total\;antimicrobials\;\left(mg\right)/DDDvetCA_{mg/kg/day}\right)}{\left(Total\;animal\;population\;\times ESVAC\;std.\;weight\;\left(kg\right)\right)} \end{array}$$

### AMR Indicators

**Frequency of resistance:** As per routine CIPARS AMR analysis (9, 23) at the isolate level, for *E. coli, Salmonella*, and *Campylobacter*, data were dichotomized into susceptible (including intermediate susceptibility) or resistant, using Clinical Laboratory Standards Institute (CLSI) breakpoints. If no CLSI interpretative criteria were available for a specific antimicrobial/bacterial combination, breakpoints were based on the distribution of MIC and harmonized with those of the United States' National Antimicrobial Resistance Monitoring System (9, 23).

**Frequency of multiclass-resistance:** The proportion of susceptible, resistant to 1 class and multiclass resistant isolates (resistant to 2–3 classes, resistant to 4–5 classes, and resistant to 6–7 classes) was determined for each bacterial species as per routine CIPARS analysis (9, 23). Resistance to ≥2 antimicrobial classes is the sum of all isolates that exhibited resistance to ≥2 classes.

**AMR Indicator Index (**AMR Ix): This is a novel AMR indicator, calculated as the percentage of resistance (or susceptibility) to a certain antimicrobial/s, adjusted by PCU (18). The AMR Ix for poultry (AMR Ix_poultry_) combines CIPARS AMR data from the broiler chickens and turkeys sampled in BC using the formula for food-producing animals described in the literature (18) and outlined in Equation 5. The organisms of interest were *Escherichia coli*, an indicator organism that is a good representative of antimicrobial exposure and the overall AMR situation (18), *Campylobacter*, a zoonotic pathogen frequently isolated from broiler chickens in Canada (36) and select organism-antimicrobial combinations specifically including those antimicrobials considered very high important and highly important to human medicine (VDD Categories I and II) (26).

**Equation 5. AMR Indicator Index calculation for poultry species sampled in British Columbia**

$$\begin{array}{l} {AMR\;Ix}_{Poultry}=\frac{R_{BrY\;\times \cdot }PCU_{BrY}}{PCU_{PoultryY}}\;+\;\frac{R_{TkY\;\times \cdot }PCU_{TkY}}{PCU_{PoultryY}}\; \end{array}$$

Where:

R_BrY_-% resistance or % fully susceptible in broiler chickens (**Table 2**); calculated for all sampled flocks, per year from 2013 to 2017.
R_TkY_-% resistance or fully susceptible in turkeys (**Table 2**); calculated per year from 2013 to 2017.
PCU_BrY_-PCU for broiler chickens; calculated per year from 2013 to 2017.
PCU_TkY_-PCU for turkeys; calculated per year from 2013 to 2017.
PCU_PoultryY_-total PCU for all poultry species; calculated per year from 2013 to 2017.
Year-specific biomass for each species is summarized in Table 1.

**Table 1:** Farm characteristics of CIPARS broiler chicken and turkey layer flocks sampled in British Columbia.

	**2013**	**2014**	**2015**	**2016**	**2017**	**Total**	**Mean**	**Std. dev**.
**BROILER CHICKENS**								
Number of flocks sampled, *n*	**26**	**30**	**25**	**32**	**30**	**143**	29	3
Population (number of birds)	522,525	650,756	592,652	765,987	732,417	3,264,336	652,867	99,679
Biomass (using 1 kg ESVAC weight)	522,525	650,756	592,652	765,987	732,417	3,264,336	652,867	99,679
Days at risk (average)	33	33	33	33	34	N/A	33	0
Milligrams active ingredients	54,512,352	67,656,030	54,790,215	73,658,806	71,972,475	322,589,877	64,517,976	9,269,757
mg/PCU	104	104	92	96	98	99	99	5
**TURKEYS**								
Number of flocks sampled, *n*	**29**	**29**	**30**	**30**	**27**	**145**	29	1
Population (number of birds)	253,930	270,750	267,228	303,641	246,046	1,341,594	268,319	22,124
Biomass (using 6.5 kg ESVAC weight)	1,650,542	1,759,872	1,736,982	1,973,663	1,599,299	8,720,358	1,744,072	143,805
Days at risk	87	84	88	88	89	N/A	87	2
Milligrams active ingredients	149,355,383	120,425,553	74,654,795	219,925,956	225,819,340	790,181,027	158,036,205	64,937,031
mg/PCU	90	68	43	111	141	91	91	38
**BROILER CHICKENS AND TURKEYS**								
Population, total	776,455	921,506	859,880	1,069,627	978,463	4,605,930	921,186	111,827
Biomass (PCU), total	2,173,067	2,410,628	2,329,634	2,739,650	2,331,716	11,984,694	2,396,939	210,084
Milligrams active ingredients, total	203,867,735	188,081,583	129,445,009	293,584,762	297,791,815	1,112,770,904	222,554,181	72,306,477
mg/PCU, total	94	78	56	107	128	93	93	27

*ESVAC, European Surveillance for Veterinary Antimicrobial Consumption; PCU, population correction unit*.

**Primary AMR Indicator Index**: AMR Ix_Susceptible *E. coli*_ was calculated as the proportion of *E. coli* isolates fully susceptible to the antimicrobials tested for by CIPARS adjusted by the PCU; this is consistent with ECDC/EFSA/EMA's primary AMR index (18).

**Secondary AMR Indicator Index**: Four secondary AMR Ix were determined: (1) AMR Ix _CRO−R *E.coli*_, was calculated as the proportion of *E. coli* isolates resistant to ceftriaxone (CRO-R) adjusted by the PCU; this AMR Ix was used instead of the ECDC/EFSA/EMA's Extended-Spectrum Beta-Lactamases (ESBL) and AmpC- producing *E coli* since these are not yet routinely tested at CIPARS (18); (2) AMR Ix_≥2__Multiclass−R *E. coli*_ calculated as the proportion of isolates resistant to ≥2 classes of antimicrobials adjusted by the PCU; this has relevance to the monitoring of the impact of overall AMU on AMR (18); (3) AMR Ix _CIP−RCampylobacter_, calculated as the proportion of *Campylobacter* isolates resistant to ciprofloxacin (CIP-R) adjusted by the PCU; the organism-antimicrobial combination is closely monitored by CIPARS due to the emerging resistance observed (36, 37), and; (4) AMR Ix _GEN−R *E.coli*_, calculated as the proportion of isolates resistant to gentamicin (GEN-R) adjusted by the PCU. This indicator was selected as CIPARS has detected an emerging increasing trend in gentamicin use and corresponding resistance in broiler chicken isolates (31). It is important to note that, for this paper, AMR Ix for ≥2 antimicrobial classes was used instead of ECDC/EFSA/EMA's resistance to AMR Ix for ≥3 antimicrobial classes due to slight differences in isolate number (i.e., little differences between the number of isolates resistant to greater than and equal to 2 antimicrobials vs. greater than and equal to 3 antimicrobials), and CIP-R *Campylobacter* was used instead of ECDC/EFSA/EMA's CIP-R *E. coli* (i.e., there were 4 broiler chicken isolates and 1 turkey CIP-R *E. coli* isolated from the CIPARS samples between 2013 and 2017) due to more robust CIP-R *Campylobacter* data.

### Integration of Poultry AMU and AMR Indicators

AMU and AMR indicators were combined into a figure to descriptively assess potential similarities in trends over time: (1) AMU frequency and AMR (% R), by species, for use of, and *E. coli* resistance to, CRO, GEN, and LINC-SPEC and (2) AMU in mg/PCU and AMR Ix for the following: (a) total AMU for broilers and turkeys across all antimicrobials (mg/PCU) and AMR Ix _Susceptible *E. coli*_ and AMR Ix_≥2__Multiclass−R *E. coli*_, (b) TIO mg/PCU and AMR Ix _CRO−R *E.coli*_, and (c) GEN and LINC-SPEC mg/PCU_poultry_ and AMR Ix _GEN−R *E.coli*._

### Descriptive and Temporal Analysis

Analysis of the data was conducted in Microsoft Excel (Office 14), Stata SE Version 15 (College Station, Texas) and SASv12.1 (Cary, North Carolina).

**AMU**. Temporal changes were determined following routine CIPARS analysis protocols (9, 23). In brief, frequency of AMU by AAI during the most recent surveillance year (2017 referent year) was compared to the initial surveillance year (2013), and the preceding year (2016) using logistic regression models (asymptotic or exact models depending on prevalence of the outcome variable). Models were developed with year as a categorical independent variable and using *P* ≤ 0.05 for significance (i.e., marked by the use of the words “significant” or “significantly” throughout the text). For the AMU indicators, data by antimicrobial class and the total of all classes per year were described. Changes in AMU indicators, between surveillance years were expressed as percent change (i.e., current year or initial surveillance year minus previous year divided by the previous year or initial surveillance year then multiplied by 100).

**AMR**. Resistance prevalence estimates were adjusted for clustering at the flock level using generalized estimating equations (GEE) with a binary outcome, logit-link function, and exchangeable correlation structure. Null binomial response models were run for each antimicrobial and from each null model, the intercept (β_0_) and 95% confidence intervals were used to calculate population-averaged prevalence estimates using the formula [1 + exp(-β_0_)]^−1^. For the temporal analysis, models were developed similar to those described above for the analysis of AMU data with year as a categorical independent variable and *P* ≤ 0.05 for significance. Temporal changes in multiclass resistance prevalence are expressed as percent change in multiclass resistance (i.e., current year or initial surveillance year minus previous year divided by the previous year or initial surveillance year then multiplied by 100).

**AMR Ix**. Change in AMR Ix, by indicators, between surveillance years are expressed as percent change (i.e., current year or initial surveillance year minus previous year divided by the previous year or initial surveillance year then multiplied by 100).

## Results

### Farm Characteristics

Farm-level characteristics during the study period are summarized in Table 1. From 2013 to 2017, a total of 143 broiler flocks (Mean: 29 flocks/year) and 145 turkey flocks (Mean: 29 flocks/year) were sampled. The total biomass (i.e., estimated based on ESVAC standard weight at treatment) was 3.6 million kg broiler chickens (Mean: 0.65 million kg/year) and 8.72 million kg turkeys (Mean: 1.7 million kg/year). The broiler chicken and turkey flocks were sampled by all the major veterinary practices in BC and the poults/chicks sampled originated from all the major hatcheries located in the province. Overall, 25% of flocks were classified as RWA or were a part of an antibiotic-free program (ABF) and organic. These flocks were not using medicated feed, including ionophores and chemical coccidiostats, from the time of chick or poult placement to the time of pre-harvest sampling.

### AMU Metrics and Indicators

#### Count Based AMU Metric: Frequency of Use

Table 2 summarizes AMU frequency by route of administration and by VDD categorization of antimicrobials (26). In broilers, the most frequently used antimicrobials in feed were bacitracin (BAC) (46%), virginiamycin (VIR) (37%), and penicillin G procaine (PEN) (28%). The frequency of VIR use decreased significantly from 54% in 2013 to 23% in 2017 (*P* ≤ 0.05). Avilamycin (AVI) was used beginning in 2014 and the frequency of use increased from 7 to 23%. The frequency of flocks not reporting any AMU in feed increased significantly, from 13% (2013) to 37% (2017) (*P* ≤ 0.05). In turkeys, the top 3 ranking antimicrobials had similar frequency to broilers: BAC (51%), VIR (40%), and PEN (5%).

**Table 2:** Antimicrobial use frequency (number of flocks reporting use/total number of flocks sampled) in CIPARS broiler chicken and turkey flocks in British Columbia, 2013-2017.

**Years**		**2013**	**2014**	**2015**	**2016**	**2017**	**All years**
**BROILERS**							
**Number of flocks**		26	30	25	32	30	143
Feed							
II	Penicillin G potassium	0%	17%	20%	0%	0%	7%
	Penicillin G procaine	50%	7%	24%	31%	30%	28%
	Virginiamycin	**54%^a^**	34%	36%	41%	**23%^a^^↓^**	37%
III	Bacitracin	50%	45%	36%	50%	50%	46%
NA	Avilamycin	0%	7%	12%	16%	23%	12%
No antimicrobials used in feed		**13%^a^**	34%	24%	25%	**37%^a^^↑^**	27%
Water							
I	Enrofloxacin	8%	0%	0%	0%	0%	1%
II	Amoxicillin	0%	0%	4%	0%	3%	1%
	Penicillin	4%	3%	0%	0%	0%	1%
	Penicillin-streptomycin	0%	0%	4%	3%	7%	3%
III	Sulfaquinoxaline	0%	3%	0%	3%	0%	1%
	Tetracycline	0%	0%	0%	0%	3%	1%
	Tetracycline-neomycin	0%	0%	0%	0%	3%	1%
	No antimicrobials used in water	88%	93%	92%	94%	90%	91%
Injections							
I	Ceftiofur	58%	7%	0%	0%	0%	12%
II	Gentamicin	12%	20%	40%	6%	17%	18%
	Lincomycin-spectinomycin	0%	0%	20%	3%	7%	6%
	No antimicrobials used at the hatchery	35%^a^	73%	40%	91%	77%^a^^↑^	65%
**TURKEYS**							
**Number of flocks**		**29**	**29**	**30**	**30**	**27**	**145**
Feed							
II	Tylosin	0%	0%	0%	0%	7%	1%
	Penicillin G potassium	0%	0%	3%	0%	0%	1%
	Penicillin G procaine	0%	21%	0%	3%	0%	5%
	Virginiamycin	**17%^a^**	38%	67%	33%	**44%^a^^↑^**	40%
	Trimethoprim-sulfadiazine	0%	0%	0%	0%	4%	1%
III	Bacitracin	69%	55%	23%	57%	52%	51%
	Chlortetracycline	3%	3%	0%	0%	4%	2%
	No antimicrobials used in feed	24%	10%	17%	13%	11%	15%
Water							
I	Enrofloxacin	0%	0%	0%	0%	4%	1%
II	Neomycin	3%	3%	0%	3%	0%	2%
	Penicillin G procaine	3%	0%	3%	10%	4%	4%
	Penicillin-streptomycin	0%	0%	0%	10%	4%	3%
III	Oxytetracycline-neomycin	0%	0%	0%	0%	4%	1%
	Tetracycline	3%	3%	0%	0%	0%	1%
	Tetracycline-neomycin	3%	3%	0%	0%	0%	1%
	No antimicrobials used in water	90%	93%	97%	87%	93%	92%
I	Ceftiofur	3%	0%	0%	0%	0%	1%
II	Gentamicin	76%	90%	73%	83%	81%	81%
	No antimicrobials used at the hatchery	21%	10%	27%	17%	19%	19%

*Roman numerals I to IV indicated categories of importance to human medicine as outlined by the Veterinary Drugs Directorate, Health Canada; N/A not applicable (no classification at the time of writing of this manuscript). Antimicrobials included in the Table are routinely reported by countries to the OIE and include those with GP properties. Please note that there were 3 broiler chicken locks with no feed/water information. Numbers may not add up to 100% as some flocks were treated with multiple antimicrobials during the grow-out cycle (feed, water, injection)*.

a *significant (P ≤ 0.05) difference between 2013 and 2017, highlighted in bold fonts. The arrows indicate the direction of the shift (increased or decreased)*.

Antimicrobials administered via water were infrequently used both in broiler chickens (1–3% overall) and turkeys (1–4%) (Table 2). Two broiler flocks (2013) and one turkey flock (2017) reported use of enrofloxacin (ENR), a fluoroquinolone which is not labeled for use in poultry in Canada (deemed extra-label use if administered in species than cattle, pigs, dogs and cats and administered in routes other than injection).

As for the injectable antimicrobials, the reported frequency of ceftiofur (TIO) use in broiler chickens decreased from 58% (2013) to 7% in 2014, with none reported from 2015 to 2017. The use of GEN was consistently reported during the study timeframe; the frequency peaked in 2015 (40%), decreased to 6% in 2016, and then increased to 17% in 2017. Lincomycin-spectinomycin was reportedly used for the first time in broilers in 2015 (20%), then in 2016 use dropped to 3%, and in 2017 it increased to 7%. Flocks not using antimicrobials at the hatchery level varied depending on the year (dropped to 40% in 2015) but between 2013 and 2017, the number of flocks significantly increased from 35 to 77% (*P* ≤ 0.05). In turkeys, there was only one flock (3%) with reported use of TIO in 2013, with no use reported from 2014 to 2017. Gentamicin was consistently used in turkey poults (73–90%). Turkey flocks not using any antimicrobials at the hatchery were generally stable over time (17–27%).

### Weight- and Dose-Based AMU Indicators

**Figure 1:**
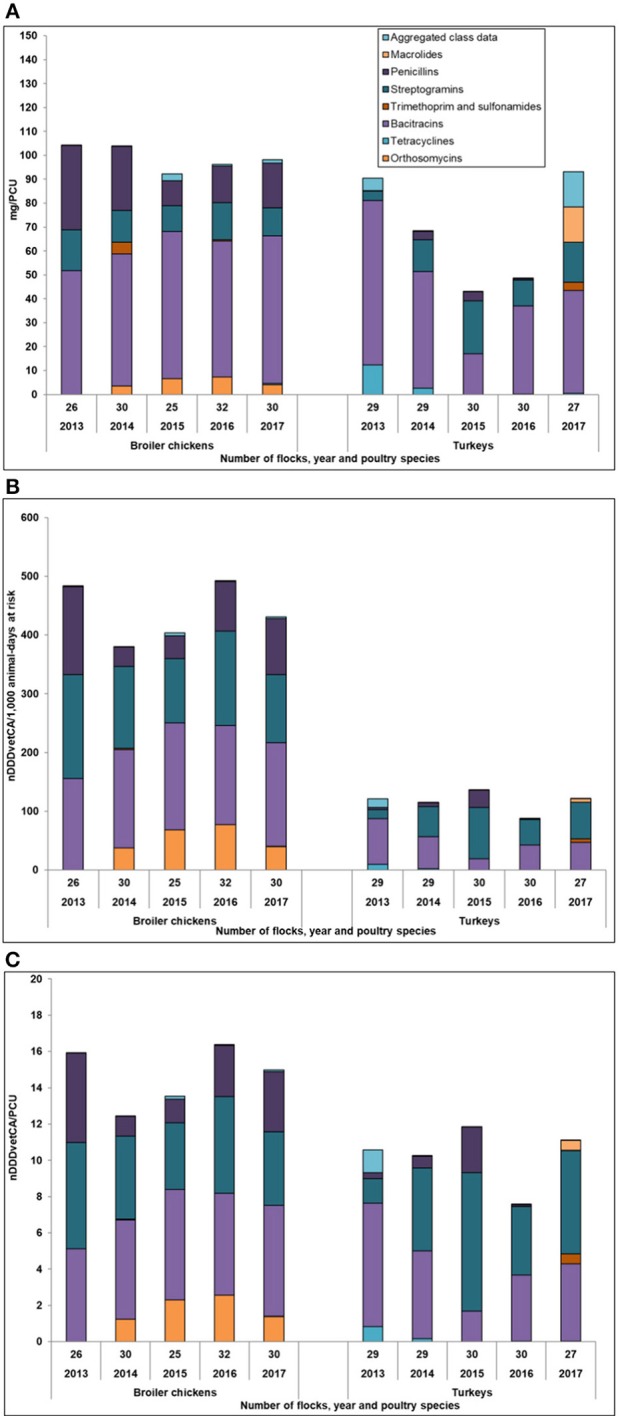
Antimicrobial use indicators comparing broiler chickens and turkeys, 2013–2017. **(A)** Milligrams/population correction unit. **(B)** Number of defined daily doses in animals/1,000 animal-days at risk. **(C)** Number of defined daily doses in animals/population correction unit. Aggregated class data was comprised of 3rd generation cephalosporins, fluoroquinolones, aminoglycosides, and lincosamides-aminocyclitols.

Figure 1 summarizes the AMU temporal trends in broiler chickens and turkeys, using different weight- and dose-based indicators. Data can be found in Annex 2.

**Weight-based (mg/PCU):** In broilers, the mg/PCU_Br_ decreased by 6% between 2013 and 2017 (104–98 mg/PCU_Br_) and during the last 2 years, it increased marginally by 2% (96–98 mg/PCU_Br_). In turkeys, the mg/PCU_Tk_ decreased by 13% between 2013 and 2017 (90–78 mg/PCU_Tk_), but during the last 2 years, 2016–2017, it increased significantly by 61% (49–78 mg/PCU). There was a decrease in mg/PCU_Tk_ between 2014 and 2015 due to the decrease in BAC use (49–17%) reported in the sampled turkey flocks. The magnitude of change between the antimicrobial classes varied depending on the year or antimicrobial class.

**Dose-based (number of DDDvetCA/1,000 animal-days at risk):** In broilers, nDDDvetCA/1,000 broiler-chicken-days at risk decreased by 11% between 2013 and 2017 (484–431 nDDDvetCA/1,000 broiler chicken days at risk), with a 13% (493–431) decrease from 2016 to 2017. As with the mg/PCU_Br_, AVI (orthosomycins) gradually increased between 2014 and 2016 but decreased in 2017. In turkeys, this AMU indicator fluctuated over time due to the shifts in the use of 3 antimicrobial classes, BAC, VIR (streptogramins), and PEN. Unlike in broiler chickens, overall, it decreased by 11% between 2013 and 2017, but remarkably increased between 2016 and 2017 by 40% (88–122 nDDDvetCA/1,000 turkey-days at risk). The increase during the last 2 years was primarily due to the increase in the use of BAC and VIR and TMPS (trimethoprim-sulfonamide) (reported for the first time in BC in 2017). Compared to broiler chickens, the yearly total values for turkeys were lower.

**Dose-based (number of DDDvetCA/PCU):** Between 2016 and 2017, the nDDDvetCA/PCU_Br_ decreased by 9% while the nDDDvetCA/PCU_Tk_ increased by 46%, but during the study timeframe (2013–2017), this indicator showed a marginal change in the total nDDvetCA/PCU [nDDvetCA/PCU_Br_: 16 to 15 (6%); nDDDvetCA/PCU_Tk_: 10.6 to 11.1 (5%)].

Figure 1 and Annex 2 summarizes the trends in the AMU indicators illustrating the inconsistencies of the temporal patterns across the 3 AMU indicators within a poultry species. In broiler chickens, mg/PCU_Br_, nDDDvetCA/1,000 broiler chicken-days at risk, and the nDDDvet/PCU_Br_ decreased between 2013 and 2017. However, during the later 2 years of the study timeframe, the mg/PCU_Br_ increased while the 2 dose-based indicators decreased. This was due to the shifts in the use of certain antimicrobial classes such as increased penicillins and bacitracins [DDDvetCA standards at 10.1 and 5.4, respectively (Annex 1)] and decreased streptogramins and orthosomycins (DDDvetCA standard of 2.9 for both) uses. In turkeys, overall, between 2013 and 2017, the mg/PCU_Tk_ decreased while the dose-based indicators increased. Between 2016 and 2017, the 3 indicators notably increased due to the shift in the use of streptogramins and bacitracins, and of macrolides (DDDvetCA standard of 26) and trimethoprim-sulfonamides (DDDvetCA standard of 10.5 and 2.2.); the latter 2 antimicrobial uses occurred in BC for the first time in 2017.

### AMR Indicators

**Figure 2:**
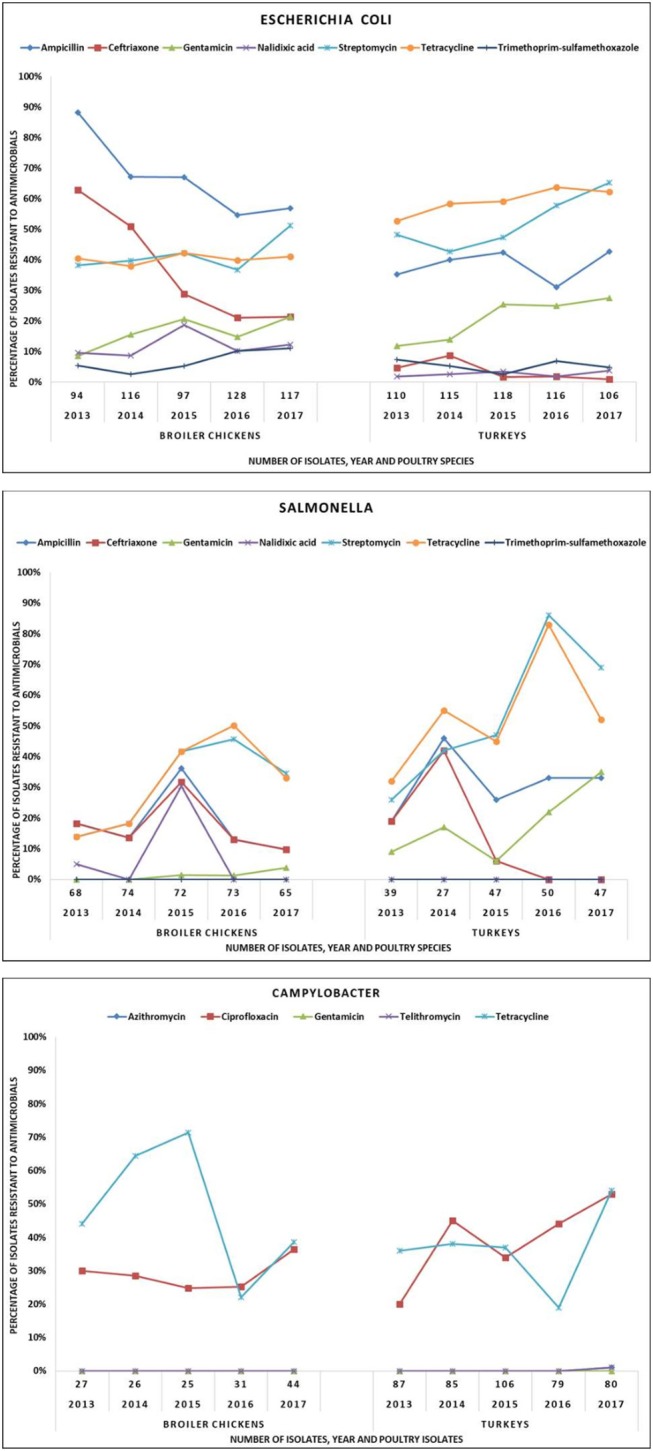
Temporal variations of resistance in *Escherichia coli, Salmonella*, and *Campylobacter* in broiler chickens and turkeys, 2013–2017.

**Frequency of resistance (%R):** The frequency of resistance to select antimicrobials in *E. coli, Salmonella*, and *Campylobacter* from the broiler chickens and turkeys are presented in Figure 2 and significant temporal changes are summarized in Annex 3. The frequency of resistance for each antimicrobial-organism combination varied by poultry species. Over time, CRO-R decreased while GEN-R increased in *E. coli* and *Salmonella* isolates and CIP-R increased in *Campylobacter* from broiler chickens and turkeys.

*Broiler chickens–E. coli:* From 2013 to 2017, resistance to ceftriaxone (CRO-R) decreased from 63 to 21%, while resistance to gentamicin (GEN-R) increased from 8 to 21%. Resistance to nalidixic acid (NAL-R) was detected in 9–19% of the isolates. Streptomycin resistance and TET-R increased significantly between 2013 and 2017.

*Broiler chickens–Salmonella:* Resistance to CRO was 18% in 2013, it decreased from 32% in 2015 (highest observed) to 10% in 2017. Nalidixic acid resistance was detected in 2013 (5%) and 2015 (30%) but no NAL-R *Salmonella* isolates were recovered in 2016 or 2017. Gentamicin resistance emerged between 2014 and 2017 (1 to 4%).

*Broiler chickens–Campylobacter:* Ciprofloxacin resistance significantly (*P* ≤ 0.05) increased between 2013 (30%) and 2017 (36%).

*Turkeys–E. coli:* Ceftriaxone resistance was generally lower than in broilers (9% peak in 2014) and remained stable (1–2%) between 2015 and 2017. Gentamicin resistance significantly (*P* ≤ 0.05) increased from 12% (2013) to 27% (2017).

*Turkeys–Salmonella:* Ceftriaxone resistance peaked at 42% in 2014 then decreased to 6% in 2015 and no resistant isolates were detected in 2016 and 2017. Gentamicin resistance fluctuated over time, but markedly increased between 2015 and 2017 (6–35%).

*Turkeys–Campylobacter:* Ciprofloxacin resistance significantly increased (*P* ≤ 0.05) from 20% in 2013 to 53% in 2017. Tetracycline resistance increased significantly (*P* ≤ 0.05) from 36% in 2013 to 54% in 2017; compared to 2016, TET-R increased significantly (*P* ≤ 0.05) from 19 to 54%.

### Multiclass Resistance

**Figure 3:**
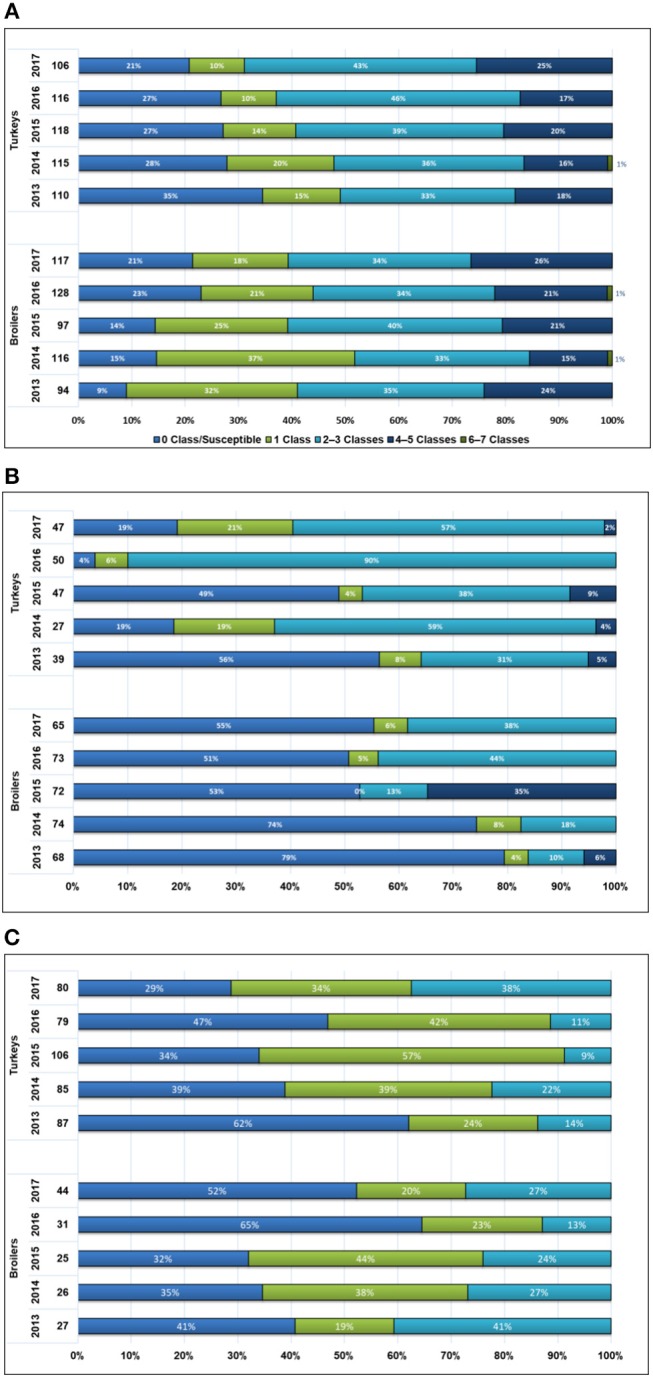
Multiclass resistance data in broiler chickens and turkeys, 2013–2017. **(A)** *Escherichia coli*, % of antimicrobial classes in resistance patterns. **(B)** *Salmonella*, % antimicrobial classes in resistance patterns. **(C)** *Campylobacter*, % antimicrobial classes in resistance pattern. As per routine CIPARS analysis (9, 23), the number of isolates by antimicrobial classes in the resistance pattern were grouped into 5 resistance patterns as follows: 0, 1, 2–3, 4–5, and 6–7.

Figure 3 demonstrates the multiclass resistance patterns of *E. coli, Salmonella*, and *Campylobacter* isolated from the 2 poultry species. Overall there were 4 *E. coli* isolates (3 from broiler chickens and 1 from turkeys) that were resistant to 6–7 antimicrobial classes detected during the study timeframe. None of the *Campylobacter* isolates were resistant to 4 or more classes of antimicrobials. The distribution of susceptible and multiclass resistant isolates varied over time across the 2 poultry species (data are presented in Annex 5). In broiler chickens, fully susceptible *E. coli* generally increased over time (9–21%), in contrast to the decreasing trend observed amongst the turkey isolates (35–21%). As for *Salmonella*, fully susceptible isolates decreased in both broiler chickens (2013: 79%, 2017: 55%) and turkeys (2013: 56%, 2017: 19%). Amongst *Campylobacter*, an increase in fully susceptible isolates was noted in broiler chickens (41–52%) while a decrease was noted in turkeys (62–29%).

### AMR Index

Results are summarized in Table 3.

**Table 3:** Antimicrobial use and resistance indicator summary, CIPARS broiler chicken and turkey flocks in British Columbia, 2013–2017.

	**2013**	**2014**	**2015**	**2016**	**2017**
**PRIMARY AMU INDICATOR^a^**					
mg/PCU_poultry_, total AMU	94	78	56	107	128
mg/PCU_poultry_, ceftiofur use	0.08	0.01	0.00	0.00	0.00
**SECONDARY AMU INDICATOR^a^**					
mg/PCU_poultry_, gentamicin and lincomycin-spectinomycin use	0.04	0.06	0.28	0.03	0.07
**PRIMARY AMR INDICATOR^a^**					
AMR Ix_Susceptible*E*. *coli*_	0.29	0.24	0.24	0.26	0.19
**SECONDARY AMR INDICATOR^b^**					
AMR Ix_CRO−RE. coli_	0.19	0.20	0.09	0.07	0.07
AMR Ix_≥2*Multiclass*−*R*E*. *coli**_	0.53	0.50	0.60	0.61	0.59
AMR Ix_CIP−RCampylobacter_	0.23	0.40	0.35	0.42	0.41
AMR Ix_GEN−R*E*. *coli*_	0.11	0.14	0.24	0.22	0.22

*AMU, antimicrobial use; AMR, antimicrobial resistance; mg/PCU, milligrams/population correction unit; mg/PCU_poultry_, the total mg/PCU in broiler chickens and turkeys combined; AMR Ix, antimicrobial resistance indicator index; CRO-R, ceftriaxone-resistant; CIP-R, ciprofloxacin-resistant; GEN-R, gentamicin-resistant; ≥2 Multiclass-R, sum of all isolates that exhibited resistance to 2 or more classes of antimicrobials*;

a,b *Please refer to the methods for the description of these indicators*.

**Primary AMR indicator:** The AMR Ix _Susceptible *E.coli*_ decreased by 34% from 0.29 in 2013 to 0.19 in 2017.

**Secondary AMR indicator:** The AMR Ix _CRO−R *E. coli*_ decreased by 61% from 0.19 in 2013 to 0.07 in 2017. During the same timeframe, the AMR Ix _≥2*Multiclass*−*R E. coli*_ increased by 12% from 0.53 in 2013 to 0.59 in 2017; AMR Ix _CIP−RCampylobacter_ increased by 79% from 0.23 to 0.41, AMR Ix _GEN−R *E.coli*_ markedly increased by 103% from 0.11 in 2013 to 0.22% in 2017.

### Integration of AMU (Frequency) and AMR Data

**Figure 4:**
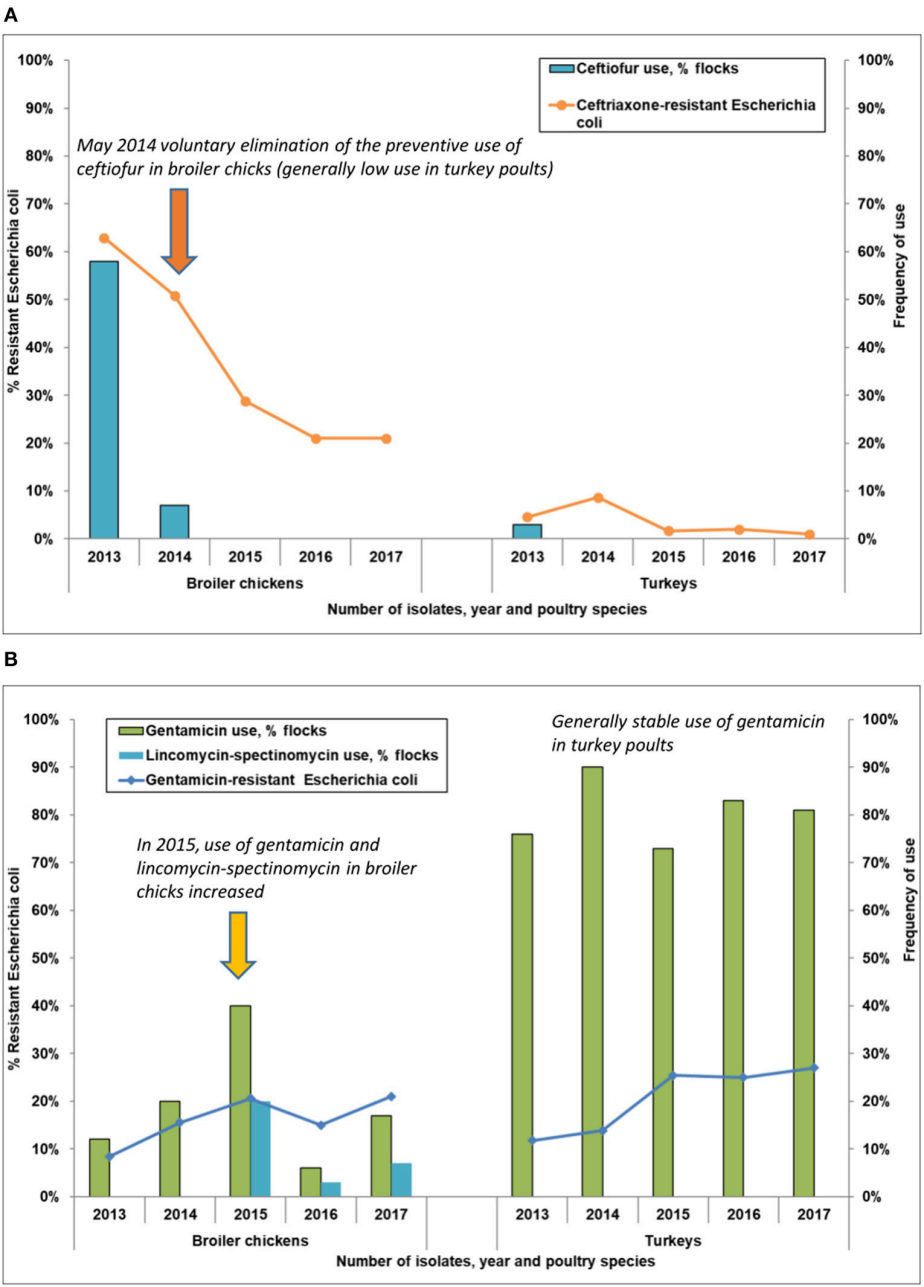
Antimicrobial use frequency and antimicrobial resistance in *Escherichia coli* in broiler chickens and turkeys. **(A)** Percentage of flocks using ceftiofur and percentage of *Escherichia coli* isolates resistant to ceftriaxone. **(B)** Percentage of flocks using gentamicin and lincomycin-spectinomycin and percentage of *Escherichia coli* isolates resistant to gentamicin.

The integration of the percentage (with low and high confidence limits) of CRO-R *E. coli* (Figure 2 and Annexes 3, 4) and the percentage of flocks that reported TIO use (Table 2) is depicted in Figure 4A. The use of TIO at the broiler hatcheries corresponded with the high CRO-R in 2013; both CRO-R and TIO use dropped in 2014, but despite no reported AMU in 2015–2017, CRO-R persisted at low level. There were few CRO-R isolates detected in turkeys (5%), which corresponded with a low TIO use (1 flock) in 2013. The relationship between the percentage of flocks that reported GEN and LINC-SPEC use and the percentage of GEN-R *E. coli* isolates is depicted in Figure 4B. The use of GEN and LINC-SPEC in broiler chicks at the hatchery corresponded with the increase in GEN-R over time. The same trend was noted in turkeys, but unlike in broiler chickens, GEN was the only antimicrobial used.

Ciprofloxacin-resistant *Campylobacter* trends for both broiler chickens and turkeys generally increased but no corresponding increase in reported ENR use was noted during the study timeframe as the use of this antimicrobial, a veterinary fluoroquinolone not approved for use in any poultry species in Canada, was relatively low (2 broiler flocks and 1 turkey flock, Table 2).

### Integration of AMU in mg/PCU and AMR Index (AMR Ix)

**Figure 5:**
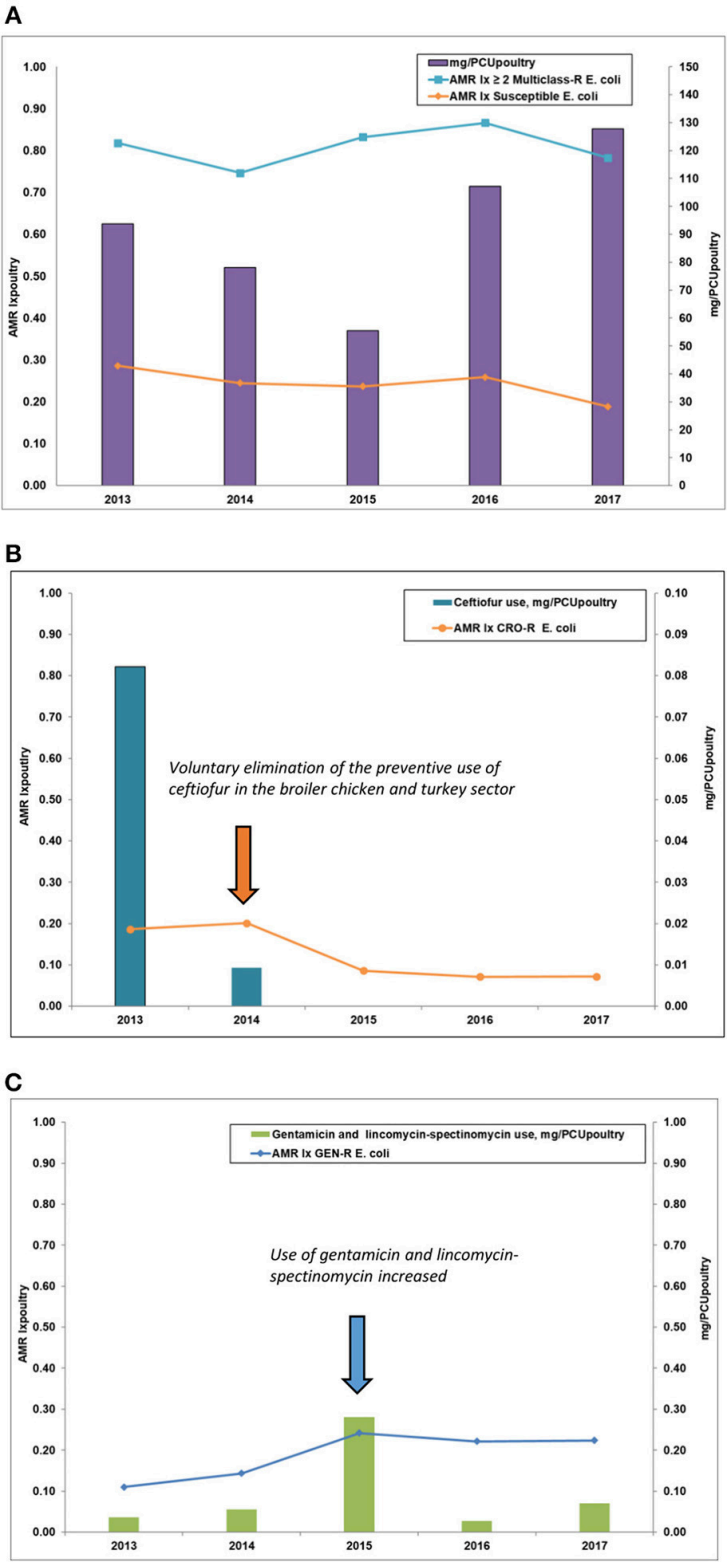
Milligrams/population correction unit (mg/PCU) and antimicrobial resistance indicator index (AMR Ix) for *Escherichia coli* in broiler chickens and turkeys. **(A)** Milligram/population correction unit_poultry_ (total use) and AMR lx (multiclass/susceptible isolates). **(B)** Milligram/population correction unit_poultrv_ (ceftiofur use) and AMR lx (ceftriaxone resistance). **(C)** Milligram/population correction unit_poultrv_ (gentamicin and lincomycin-spectinomycin use) and AMR (gentamicin resistance). AMR, antimicrobial resistance; mg/PCU_poultry_, the total milligrams/population correction unit in broiler chickens and turkeys combined; AMR Ix, antimicrobial resistance indicator index; CRO-R, ceftriaxone-resistant; GEN-R, gentamicin-resistant, ≥2 Multiclass-R, sum of all isolates that exhibited resistance to 2 or more classes of antimicrobials.

The findings are summarized in Table 3 and the relationship between AMU and AMR Ix is demonstrated in Figure 5. During the study timeframe (2013–2017), the total mg/PCU for broiler chickens and turkeys increased (94–128) which corresponded to the decrease in AMR Ix _Susceptible *E. coli*_ (0.29–0.19, decreased by 34%). However, AMR Ix _≥2*Multiclass*−*R E. coli*_paralleled the trends in total mg/PCU between 2013 and 2016 (increased) but dropped by 10% between 2016 (0.61) and 2017 (0.59). Figure 5 also depicts the relationship between the TIO use in mg/PCU_poultry_ and the AMR Ix _CRO−R *E.coli*._ The TIO mg/PCU_poultry_ decreased from 0.08 in 2013 to 0.01 in 2014, and no TIO use was reported from 2015 to 2017. A corresponding decrease in AMR Ix _CRO−R *E.coli*_ was noted over time as previously described (Table 3) however, it is important to note that despite no reported TIO use since 2014, the AMR Ix _CRO−R *E.coli*_ persisted until 2017. Also in Figure 5, the highest reported GEN and LINC-SPEC use was recorded in 2015 at 0.28 mg/PCU and decreased in 2016 (0.03 mg/PCU_poultry_) to 2017 (0.07 mg/PCU_poultry_) (Table 3) but there was no corresponding decrease in AMR Ix _GEN−R *E.coli*_ from 0.24 in 2015 (peak of use) to 0.22 in 2016 and 2017.

## Discussion

This paper described temporal trends in AMU and AMR in poultry flocks, compared AMU and AMR between the poultry species, and evaluated the effects of various AMU and AMR units of measurement for reporting flock-level data on AMU and AMR. The farm-level data provided a comprehensive overview of AMU in the broiler chicken and turkey sectors within the province in Canada. Our data showed that the direction and magnitude of either trends or discrepancies between populations could change based on the metric or indicator chosen, but when the indicators were applied simultaneously to the same dataset, multiple study objective/s described above could be achieved. Hence, we believe there is much value to a comprehensive reporting of AMU data using different indicators.

The count-based measurement (frequency of flocks medicated), when used by itself in a surveillance program, can detect changes over time; indicate how extensively on antimicrobial is used; detect shifts in AMU, particularly the shifts in the use of antimicrobials with higher importance to human medicine to antimicrobials with lower importance to human medicine or vice versa; and highlight the proportion of farms not using antimicrobials, which can be an indication of the changing production profile (i.e., increase in the number of farms raising ABF/RWA or organic birds). In Canada, frequency data have been used in studies and risk assessments exploring the link between AMU and AMR, for example, the use of TIO in poultry and AMR among *Salmonella* Heidelberg and *Escherichia coli* (38).

The weight-based indicator, mg/PCU, is becoming one of the most frequently used AMU indicators internationally (39). In the Canadian poultry industry, total population and farm-level efficiency data including daily and cumulative mortalities can be accessed from farm records as per the on-farm food safety program requirements (34, 35), feed mill delivery receipts and prescription data for medicated feed. As such, this indicator makes use of farm data already collected. The utility of this weight-based indicator for monitoring AMU during the early implementation phase of a national AMU surveillance program has been cited in the literature to quantify overall national level use (39, 40). In Europe, this indicator consistently showed a statistically significant negative association between AMU and susceptible isolates, and thus was cited as “the most adequate indicator” to monitor the impact of AMU reduction (17, 18).

The dose-based indicator, nDDDvetCA adjusted by animal time (1,000 animal-days at risk), or biomass (PCU) is currently used by 8/16 countries participating in the AACTING network. It was also cited for animal AMU reporting in the European Union/European Economic Area (12). The DDDvet is a technical unit of measurement that, unlike other indicators, account for dosing differences between active ingredients, formulations, and animal species (41).

All three AMU indicators used; mg/PCU, nDDDvetCA/1,000 animal-days at risk, and nDDDvetCA/PCU detected temporal AMU changes (i.e., total AMU and by class). The reduction in total AMU (mg/PCU) could indicate early industry actions in anticipation of the forthcoming changes in industry policies (24, 25). The magnitude of change over time varied depending on the antimicrobial class, poultry species and year. The increase noted in the dose-based indicators, specifically from 2016 to 2017, is an indication of the changes in antimicrobial preferences (i.e., switch from antimicrobials with higher daily doses to those with lower daily doses), illustrating the value of this indicator over non-dose-based indicators. As we described previously (31), the DDDvetCA assignment (Annex 1) involved stratification by route of administration (e.g., feed, water, and injection); this methodology was modified from ESVAC DDDvet assignment principles (41), where only one DDDvetCA was assigned for any oral formulation. This greater stratification of DDDvetCA assignment could be used for detecting shifts in use from one route of administration to another, such as potential shift from feed uses (i.e., preventive doses, prolonged days of administration) to water uses (i.e., treatment doses and shorter days of administration). With the anticipated changes in disease prevalence (e.g., necrotic enteritis) in the broiler chicken and turkey sectors following the implementation of the second and third steps of the industry AMU strategy (i.e., elimination of the preventive use of VDD Category II and III antimicrobials which are largely used for managing necrotic enteritis in the field), this indicator will permit detection of the shift in AMU as described above.

The three indicators enabled inter-species AMU comparison. In Europe, the mg/PCU indicator is deemed the primary AMU indicator for national sales and distribution data to monitor overall impact of AMU policy change due to its ability to detect changes in AMU over time and robustness in describing the animal population exposed (18). With the use of mg/PCU, mg/PCU_Tk_ AMU was generally lower compared to mg/PCU_Br_. The practice of feeding unmedicated rations during the late growing to finishing stages of the production period in turkeys is potentially driving the lower AMU. When nDDDvetCA/1,000 animal-days at risk was used, the magnitude of the difference between turkeys and broiler chickens was greater than the mg/PCU. This may be due to the relatively larger number of days required to achieve marketing weight for some categories, such as heavy tom turkeys. The turkey and broiler chicken sectors of the Canadian poultry industry collectively agreed to eliminate the preventive use of VDD Category II antimicrobials by the end of 2018 and VDD Category III antimicrobials by the end of 2019 (25). Additionally, by the end of 2018, the use of VDD Categories I to III antimicrobials requires veterinary oversight or to be used only with a veterinary prescription (28). It is therefore expected that the quantities reported via the surveillance data will decrease over time across all three indicators.

For AMR reporting, the percentage of isolates resistant (% R) to a specific antimicrobial is a standardized unit of measurement used internationally for reporting resistance prevalence from animals and humans. This complements trends in AMU data to assess the impact of an AMU reduction strategy (42, 43).

The AMR Ix is a new indicator that combines the frequency of resistance across the host species under surveillance, while accounting for the relative contribution of each of the host species on the overall AMR Ix (18). In this study, the antimicrobials selected for AMR Ix estimation for *E. coli* were based on their relevance to overall AMU selection pressure and their importance to human medicine (18). A zoonotic pathogen, *Campylobacter*, was included to monitor the temporal changes in CIP-R; one of the emerging antimicrobial-resistant strains of high interest in Canada that is closely monitored by CIPARS (9, 36, 37). *Salmonella* is another food-borne pathogen in Canada (44), but because of serovar variations in resistance among *Salmonella* and the unique spectrum of serovars detected by species, we did not estimate an AMR Ix for this zoonotic pathogen. Reduction targets for AMR in Canada have not been established; however, the AMR Ix could be used to monitor AMU reduction strategies, and potentially for monitoring the progress of the implementation of the industry interventions to reduce AMR.

In this study, the AMR Ix indicated success of an industry intervention and simultaneously, the unintended consequence of the intervention (i.e., AMR Ix _CRO−REcoli_ decreasing trend and AMR Ix _GEN−REcoli_ increasing trend, indicative of the shift for treating infections in young chicks from TIO to GEN). The AMR Ix is also a good complement to mg/PCU in monitoring the overall impact of changes in antimicrobial usage patterns as demonstrated in the integrated figures, such as the parallel trends noted between mg/PCU_poultry_ and the AMR Ix_≥2Multiclass−*R E.coli*_ or the disparate trends observed between mg/PCU_poultry_ and AMR Ix _Susceptible *E. coli*._ The latter observation is consistent with the literature (i.e., a consistent statistically significant association between total AMU and susceptible isolates) (18). Further analysis of the relationship between these trends in AMU and AMR indicators is required in order to investigate their statistical significance when additional risk factors for AMR are considered. When more robust data from the CIPARS program becomes available (e.g., ongoing data collection from all relevant livestock and poultry species), associations among indicators could be further assessed to substantiate the findings presented in this paper.

Surveillance data indicated that 8% of broiler flocks in 2013 and 4% of turkey flocks in 2017 reported fluoroquinolone use, with no use from 2014 to 2016. In BC in 2014, ~38 kg of fluoroquinolones were distributed by BC livestock and poultry veterinarians (45), however, the actual kg of fluoroquinolones distributed and used in poultry production is unknown. The frequency of CIP-R in *Campylobact*er remained moderately high and the reason for the persistence in Canadian flocks is unclear and ongoing monitoring and research is needed to determine the main drivers of CIP-R in poultry. The literature indicates that mutation in the Thr-86-Ile mutation in gyrA is directly assocaited with the enhanced fitness of fluoroquinolone-resistant *Campylobacter* (46). This may explain the persistency of fluoroquinolone-resistant *Campylobacter* in the absence of antimicrobial selection pressure in poultry and their environment (47, 48). Changes in the Thr-86-Ile in the gyrA among CIP-R *Campylobacter* recovered from broiler chickens and turkeys in BC needed to be characterized. Vertical transmission and other on-farm sources of contamination (i.e., between farms, other livestock species) and farm-level risk factors such as insufficient downtime/rest period, cleaning, and disinfection have been hypothesized as potential on-farm sources (48) and also warrant investigation.

As described in our previous analysis (31), AMU data were collected from a single grow-out cycle. These data cannot be extrapolated to 1 year of production (e.g., 6 grow-out cycle for broilers and 3–5 cycles for turkeys) due to variations in seasonal antimicrobial use, bird populations, and antimicrobial options (e.g., new products approved for use in poultry). In addition, due to regional differences in disease pressures, agriculture profile variations between provinces (predominant food animal production species), and other operational factors that could potentially drive AMU, these poultry-specific provincial data cannot be extrapolated to national level results. Despite these limitations, data available at the time of writing of this report permitted exploration of various AMU indicators for detecting trends in AMU and AMR and their utility for future data integration. As the program is progressively strengthened (i.e., by expansion of the farm program in other species such as layers, beef and dairy cattle), robust data could be generated which will subsequently improve the precision of our AMR Ix estimates.

No single AMU or AMR indicator can meet all possible surveillance objectives. Different indicators, in isolation or when integrated with others, highlight different aspects of the complex AMU/AMR issue. Choosing appropriate indicators and then applying them appropriately requires careful consideration of both the data available and the desired objectives. Our findings highlight the utility of AMR Ix in monitoring changes in AMR of organisms of interest to the animal and public health sectors. For integration to be meaningful, data collection, sampling, laboratory techniques, and data management across all sectors must be harmonized. As in any other surveillance program (18), multi-species data may not be available consistently from year to year due to limited resources, which would impact temporal and inter-sectoral analyses. The integration of AMR Ix and an AMU indicator (e.g., mg/PCU) aids in monitoring the effect of AMU reduction interventions, such as the elimination of preventive use of certain antimicrobials (e.g., TIO and ENR which were historically used in an extra-label manner in poultry in Canada), reduction in the use of hatchery-administered antimicrobials (e.g., GEN and LINC-SPEC), or increasing participation in ABF/RWA and organic production (i.e., no use or use of non-medically-important antimicrobials). This study highlights the importance of an ongoing farm-level data AMU and AMR surveillance for monitoring the impact of industry and government interventions to reduce AMR and to inform enhancements to other existing on-farm food safety, flock health management, and AMU practices (e.g., extra-label drug use reduction).

## Author Contributions

AA, SG, DFL, and CC conceived the study. RI and RR-S supervised all surveillance components. AA, SG, DFL, and AD validated, analyzed, and interpreted the data. AB, DL, DFL, and CC conceptualized and developed the DDDvetCA standards. AA prepared the initial draft, figures, tables, and appendices. All authors contributed to the writing and editing of the manuscript.

### Conflict of Interest Statement

The authors declare that the research was conducted in the absence of any commercial or financial relationships that could be construed as a potential conflict of interest.
