# Supplementary material for: Antimicrobial Use and Antimicrobial Resistance Indicators—Integration of Farm-Level Surveillance Data From Broiler Chickens and Turkeys in British Columbia, Canada
Source: Front Vet Sci. 2019 May 3;6:131. doi: 10.3389/fvets.2019.00131 (PMC6509235; doi:10.3389/fvets.2019.00131)
Supplement: Supplementary file 1 [file Table_1.DOCX]

ANNEX 1 | Canadian Defined Daily Doses for animals (DDDvetCA) standard values for broiler chickens and turkeys

| **Route of administration** | **European route of administration** | **Antimicrobial** | **Average dose basis** | **Average dose** | **DDDvetCA (mg_drug_/kg_animal_/day)** |
| --- | --- | --- | --- | --- | --- |
| Injectable | Parenteral | Ceftiofur (ELDU) | T, P | 2.6 | 2.6 |
|  |  | Gentamicin | T, P | 10.8 | 10.8 |
|  |  | Lincomycin-spectinomycin^a^ (ELDU) | T, P | 6.0 | 6.0 |
|  |  | Spectinomycin-lincomycin^a^ (ELDU) | T, P | 12.0 | 12.0 |
| Feed | Oral | Avilamycin | T, P | 22.5 | 2.9 |
|  |  | Bacitracin | T, P | 77.9 | 10.1 |
|  |  | Chlortetracycline | T, P | 128.3 | 16.7 |
|  |  | Erythromycin | T, P | 220.0 | 28.6 |
|  |  | Oxytetracycline | T, P | 128.3 | 16.7 |
|  |  | Procaine Penicillin G | T, P | 41.3 | 5.4 |
|  |  | Sulfadiazine-trimethoprim^a^ (ELDU) | T, P | 83.3 | 10.8 |
|  |  | Trimethoprim-sulfadiazine^a^ (ELDU) | T, P | 16.8 | 2.2 |
|  |  | Tylosin | T, P | 200.0 | 26.0 |
|  |  | Virginiamycin | T, P | 22.0 | 2.9 |
| Water | Oral | Amoxicillin | T, P | 52.0 | 12.0 |
|  |  | Apramycin (ELDU) | T, P | 100.0 | 23.0 |
|  |  | Enrofloxacin (ELDU) | T, P | 25.0 | 5.8 |
|  |  | Erythromycin | T, P | 86.7 | 19.9 |
|  |  | Lincomycin | T, P | 16.0 | 3.7 |
|  |  | Lincomycin-spectinomycin^a^ | T, P | 277.5 | 63.8 |
|  |  | Neomycin | T, P | 94.8 | 21.8 |
|  |  | Oxytetracycline | T, P | 81.9 | 18.8 |
|  |  | Pennicillin G | T, P | 178.3 | 41.0 |
|  |  | Penicillin G (supp) | T, P | 16.5 | 3.8 |
|  |  | Spectinomycin-lincomycin^a^ | T, P | 555.0 | 127.7 |
|  |  | Streptomycin (supp) | T, P | 85.2 | 19.6 |
|  |  | Sulfamethazine | T, P | 1027.8 | 236.4 |
|  |  | Sulfaquinoxaline | T, P | 317.2 | 72.9 |
|  |  | Sulfaquinoxaline-pyrimethamine^a^ | T, P | 48.8 | 11.2 |
|  |  | Tetracycline | T, P | 93.1 | 21.4 |
|  |  | Tylosin | T, P | 312.5 | 71.9 |

*Extra-label drug use (ELDU) poultry, dose, or doses were derived from expert opinion or veterinary consultations; Supp = supplement or product has lower level of drug; Average dose = average of all doses indicated in available products listed in the Compendium of Medicating Ingredients Brochure (*[*http://www.inspection.gc.ca/animals/feeds/medicating-ingredients/eng/1300212600464/1320602461227*](http://www.inspection.gc.ca/animals/feeds/medicating-ingredients/eng/1300212600464/1320602461227)*) and Compendium of Veterinary Products (*[*https://bam.cvpservice.com/*](https://bam.cvpservice.com/) *); values were multiplied to the standard values for either feed or water intake to obtain the DDDvetCA standard for poultry (*[*http://publications.gc.ca/collections/collection_2018/aspc-phac/HP2-4-2016-eng.pdf*](http://publications.gc.ca/collections/collection_2018/aspc-phac/HP2-4-2016-eng.pdf)*);*

*DDDvetCA = Canadian Defined Daily Doses for animals (average labelled dose) in milligrams per kilogram broiler chicken or turkey per day (mg_drug_/kg_animal_/day) .*

*P – prevention*

*T - treatment*

*a Antimicrobials with hyphen is a combination drug; the values for this row pertain to the first drug in the sequence*
