# Supplementary material for: Antimicrobial Use and Antimicrobial Resistance Indicators—Integration of Farm-Level Surveillance Data From Broiler Chickens and Turkeys in British Columbia, Canada
Source: Front Vet Sci. 2019 May 3;6:131. doi: 10.3389/fvets.2019.00131 (PMC6509235; doi:10.3389/fvets.2019.00131)
Supplement: Supplementary file 2 [file Table_2.DOCX]

**ANNEX 2** | Data for Figure 1 - antimicrobial use indicators

|  |  | **Broilers** |  |  |  |  |  | **Turkeys** |  |  |  |  |
| --- | --- | --- | --- | --- | --- | --- | --- | --- | --- | --- | --- | --- |
|  |  | **2013** | **2014** | **2015** | **2016** | **2017** |  | **2013** | **2014** | **2015** | **2016** | **2017** |
|  |  | **24** | **29** | **25** | **32** | **30** |  | **29** | **29** | **30** | **30** | **27** |
| **mg/PCU** |  |  |  |  |  |  |  |  |  |  |  |  |
| I | 3rd generation cephalosporins | 0.08 | 0.01 | 0.00 | 0.00 | 0.00 |  | 0.0 | 0.0 | 0.0 | 0.0 | 0.0 |
|  | Fluoroquinolones | 0.04 | 0.00 | 0.00 | 0.00 | 0.00 |  | 0.0 | 0.0 | 0.0 | 0.0 | 0.0 |
| II | Aminoglycosides | 0.04 | 0.06 | 2.55 | 0.67 | 1.47 |  | 5.3 | 0.3 | 0.1 | 0.4 | 0.1 |
|  | Lincosamides-aminocyclitols | 0.00 | 0.00 | 0.20 | 0.02 | 0.04 |  | 0.0 | 0.0 | 0.0 | 0.0 | 0.0 |
|  | Macrolides | 0.00 | 0.00 | 0.00 | 0.00 | 0.00 |  | 0.0 | 0.0 | 0.0 | 0.0 | 14.8 |
|  | Penicillins | 35.28 | 26.93 | 10.56 | 15.21 | 18.70 |  | 0.2 | 3.5 | 3.7 | 0.5 | 0.0 |
|  | Streptogramins | 16.93 | 13.23 | 10.64 | 15.48 | 11.71 |  | 3.9 | 13.3 | 22.2 | 10.9 | 16.6 |
|  | Trimethoprim and sulfonamides | 0.00 | 4.82 | 0.00 | 0.58 | 0.00 |  | 0.0 | 0.0 | 0.0 | 0.0 | 3.5 |
| III | Bacitracins | 51.85 | 55.29 | 61.56 | 56.78 | 61.75 |  | 68.7 | 48.8 | 17.0 | 36.8 | 43.0 |
|  | Tetracyclines | 0.00 | 0.00 | 0.00 | 0.00 | 0.59 |  | 12.3 | 2.7 | 0.0 | 0.2 | 0.4 |
| N/A | Orthosomycins | 0.00 | 3.57 | 6.67 | 7.40 | 4.01 |  | 0.0 | 0.0 | 0.0 | 0.0 | 0.0 |
|  | **Total** | **104.21** | **103.90** | **92.17** | **96.13** | **98.26** |  | **90.5** | **68.4** | **43.0** | **48.7** | **78.5** |
| **nDDDvetCA/1,000 animal-days at risk** | |  |  |  |  |  |  |  |  |  |  |  |
| I | Aminoglycosides | 0.10 | 0.16 | 3.97 | 1.03 | 2.58 |  | 14.17 | 0.55 | 0.13 | 0.62 | 0.15 |
|  | Fluoroquinolones | 0.20 | 0.00 | 0.00 | 0.00 | 0.00 |  | 0.00 | 0.00 | 0.00 | 0.00 | 0.00 |
|  | Third generation cephalosporins | 0.96 | 0.11 | 0.00 | 0.00 | 0.00 |  | 0.01 | 0.00 | 0.00 | 0.00 | 0.00 |
| II | Lincosamides-aminocyclitols | 0.00 | 0.00 | 0.67 | 0.06 | 0.14 |  | 0.00 | 0.00 | 0.00 | 0.00 | 0.00 |
|  | Macrolides | 0.00 | 0.00 | 0.00 | 0.00 | 0.00 |  | 0.00 | 0.00 | 0.00 | 0.00 | 6.21 |
|  | Penicillins | 149.56 | 33.05 | 39.39 | 84.98 | 95.50 |  | 3.88 | 7.18 | 28.79 | 1.24 | 0.01 |
|  | Streptogramins | 177.09 | 139.45 | 109.30 | 160.52 | 116.08 |  | 15.52 | 51.40 | 87.49 | 43.52 | 62.42 |
|  | Trimethoprim-sulfonamides | 0.00 | 2.02 | 0.00 | 0.24 | 0.00 |  | 0.00 | 0.00 | 0.00 | 0.00 | 5.92 |
| III | Bacitracins | 155.76 | 167.27 | 181.62 | 169.02 | 175.78 |  | 77.82 | 54.22 | 19.24 | 41.98 | 46.59 |
|  | Tetracyclines | 0.00 | 0.00 | 0.00 | 0.00 | 0.77 |  | 9.43 | 1.96 | 0.00 | 0.28 | 0.26 |
| N/A | Orthosomycins | 0.00 | 37.56 | 68.49 | 76.69 | 39.75 |  | 0.00 | 0.00 | 0.00 | 0.00 | 0.00 |
|  | **Total** | **483.68** | **379.61** | **403.45** | **492.54** | **430.60** |  | **120.83** | **115.31** | **135.66** | **87.64** | **121.56** |
| **nDDDvetCA/PCU** | |  |  |  |  |  |  |  |  |  |  |  |
| I | Fluoroquinolones | 0.00 | 0.00 | 0.00 | 0.00 | 0.00 |  | 0.00 | 0.00 | 0.00 | 0.00 | 0.00 |
| I | Third generation cephalosporins | 0.00 | 0.00 | 0.00 | 0.00 | 0.00 |  | 0.00 | 0.00 | 0.00 | 0.00 | 0.00 |
| II | Aminoglycosides | 0.00 | 0.00 | 0.10 | 0.00 | 0.10 |  | 1.20 | 0.00 | 0.00 | 0.10 | 0.00 |
| II | Lincosamides-aminocyclitols | 0.00 | 0.00 | 0.00 | 0.00 | 0.00 |  | 0.00 | 0.00 | 0.00 | 0.00 | 0.00 |
| II | Macrolides | 0.00 | 0.00 | 0.00 | 0.00 | 0.00 |  | 0.00 | 0.00 | 0.00 | 0.00 | 0.60 |
| II | Penicillins | 4.90 | 1.10 | 1.30 | 2.80 | 3.30 |  | 0.30 | 0.60 | 2.50 | 0.10 | 0.00 |
| II | Streptogramins | 5.80 | 4.60 | 3.70 | 5.30 | 4.00 |  | 1.40 | 4.60 | 7.60 | 3.80 | 5.70 |
| II | Trimethoprim and sulfonamides | 0.00 | 0.10 | 0.00 | 0.00 | 0.00 |  | 0.00 | 0.00 | 0.00 | 0.00 | 0.50 |
| III | Bacitracins | 5.10 | 5.50 | 6.10 | 5.60 | 6.10 |  | 6.80 | 4.80 | 1.70 | 3.60 | 4.30 |
| III | Tetracyclines | 0.00 | 0.00 | 0.00 | 0.00 | 0.00 |  | 0.80 | 0.20 | 0.00 | 0.00 | 0.00 |
| N/A | Orthosomycins | 0.00 | 1.20 | 2.30 | 2.60 | 1.40 |  | 0.00 | 0.00 | 0.00 | 0.00 | 0.00 |
|  | **Total** | **15.90** | **12.40** | **13.50** | **16.40** | **15.00** |  | **10.60** | **10.30** | **11.90** | **7.60** | **11.10** |
