# Supplementary material for: Antimicrobial Use and Antimicrobial Resistance Indicators—Integration of Farm-Level Surveillance Data From Broiler Chickens and Turkeys in British Columbia, Canada
Source: Front Vet Sci. 2019 May 3;6:131. doi: 10.3389/fvets.2019.00131 (PMC6509235; doi:10.3389/fvets.2019.00131)
Supplement: Supplementary file 3 [file Table_3.DOCX]

**ANNEX 3 | Resistance in *Escherichia coli, Salmonella* and *Campylobacter* from CIPARS broiler chicken and turkey flocks in British Columbia Canada, 2013-2017**

|  | | **Broiler chickens** | | | | |  | **Turkeys** | | | | |
| --- | --- | --- | --- | --- | --- | --- | --- | --- | --- | --- | --- | --- |
| **Year** |  | **2013** | **2014** | **2015** | **2016** | **2017** |  | **2013** | **2014** | **2015** | **2016** | **2017** |
| **Number of *E. coli* isolates** | | **94** | **116** | **97** | **128** | **117** |  | **110** | **115** | **118** | **116** | **106** |
|  | **% of isolates resistant** | |  |  |  |  |  |  |  |  |  |  |
|  | Ampicillin | 88% | 67% | 67% | 55% | 57% |  | 35% | 40% | 42% | 31% | 43% |
|  | Ceftriaxone | 63% | 51% | 29% | 21% | 21% |  | 5% | 9% | 2% | 2% | 1% |
|  | Gentamicin | 8% | 16% | 21% | 15% | 21% |  | **12%^a^** | 14% | 25% | 25% | **27%^a↑^** |
|  | Nalidixic acid | 10% | 9% | 19% | 10% | 12% |  | 2% | 3% | 3% | 2% | 4% |
|  | Streptomycin | **38%^a^** | 40% | 42% | 37% | **51%^a↑^** |  | 48% | 43% | 47% | 58% | 65% |
|  | Tetracycline | **40%^a^** | 38% | 42% | 40% | **41%^a↑^** |  | 53% | **58%^a^** | 59% | 64% | **62%^a↑^** |
|  | Trimethoprim-sulfamethoxazole | 5% | 3% | 5% | 10% | 11% |  | 7% | 5% | 3% | 7% | 5% |
| **Number of *Salmonella* isolates** | | **68** | **74** | **72** | **73** | **65** |  | **39** | **27** | **47** | **50** | **47** |
|  | **% of isolates resistant** | |  |  |  |  |  |  |  |  |  |  |
|  | Ampicillin | 18% | 14% | 36% | 13% | 10% |  | 19% | 46% | 26% | 33% | 33% |
|  | Ceftriaxone | 18% | 14% | 32% | 13% | 10% |  | 19% | 42% | 6% | 0% | 0% |
|  | Gentamicin | 0% | 0% | 1% | 1% | 4% |  | 9% | 17% | 6% | 22% | 35% |
|  | Nalidixic acid | 5% | 0% | 30% | 0% | 0% |  | 0% | 0% | 0% | 0% | 0% |
|  | Streptomycin | 14% | 18% | 42% | 46% | 35% |  | 26% | 42% | 47% | 86% | 69% |
|  | Tetracycline | 14% | 18% | 42% | 50% | 33% |  | 32% | 55% | 45% | 83% | 52% |
|  | Trimethoprim-sulfamethoxazole | 0% | 0% | 0% | 0% | 0% |  | 0% | 0% | 0% | 0% | 0% |
| **Number of *Campylobacter* isolates** | | **27** | **26** | **25** | **31** | **44** |  | **87** | **85** | **106** | **79** | **80** |
|  | **% of isolates resistant** | |  |  |  |  |  |  |  |  |  |  |
|  | Azithromycin | 0% | 0% | 0% | 0% | 0% |  | 0% | 0% | 0% | 0% | 1% |
|  | Ciprofloxacin | **30%^a^** | 29% | 25% | 25% | **36%^a↑^** |  | 20% | **45%^a^** | 34% | 44% | **53%^a↑^** |
|  | Gentamicin | 0% | 0% | 0% | 0% | 0% |  | 0% | 0% | 0% | 0% | 0% |
|  | Telithromycin | 0% | 0% | 0% | 0% | 0% |  | 0% | 0% | 0% | 0% | 1% |
|  | Tetracycline | 44% | 64% | 71% | 22% | 39% |  | 36% | 38% | 37% | **19%^b^** | **54%^b↑^** |

*^a^significant (P≤0.05) difference between 2013 and 2017.*

*^b^significant (P≤0.05) difference between 2016 and 2017.*

*Please note that there were 3 broiler flocks with missing feed and water antimicrobial use information.*

*For the temporal analysis, the frequency of antimicrobial use and antimicrobial resistance during the most recent surveillance year (2017 referent year) was compared to the initial surveillance year (2013), and the preceding year (2016) using logistic regression models (asymptotic or exact models depending on prevalence of the outcome variable). Models were developed with year as a categorical independent variable and using P ≤0.05 for significance*
