# Supplementary material for: Antimicrobial Use and Antimicrobial Resistance Indicators—Integration of Farm-Level Surveillance Data From Broiler Chickens and Turkeys in British Columbia, Canada
Source: Front Vet Sci. 2019 May 3;6:131. doi: 10.3389/fvets.2019.00131 (PMC6509235; doi:10.3389/fvets.2019.00131)
Supplement: Supplementary file 5 [file Table_5.DOCX]

**ANNEX 5** | Data for Figure 3 - Number of isolates, by the number of antimicrobial classes in the resistance pattern

|  | **Broilers** | | | | |  | **Turkeys** | | | | |
| --- | --- | --- | --- | --- | --- | --- | --- | --- | --- | --- | --- |
|  | **2013** | **2014** | **2015** | **2016** | **2017** |  | **2013** | **2014** | **2015** | **2016** | **2017** |
| ***E. coli*** | **94** | **116** | **97** | **128** | **117** |  | **110** | **115** | **118** | **116** | **106** |
| 0 Class/Susceptible | 9% | 15% | 14% | 23% | 21% |  | 35% | 28% | 27% | 27% | 21% |
| 1 Class | 32% | 37% | 25% | 21% | 18% |  | 15% | 20% | 14% | 10% | 10% |
| 2–3 Classes | 35% | 33% | 40% | 34% | 34% |  | 33% | 36% | 39% | 46% | 43% |
| 4–5 Classes | 24% | 15% | 21% | 21% | 26% |  | 18% | 16% | 20% | 17% | 25% |
| 6–7 Classes | 0% | 1% | 0% | 1% | 0% |  | 0% | 1% | 0% | 0% | 0% |
|  | 100% | 100% | 100% | 100% | 100% |  | 100% | 100% | 100% | 100% | 100% |
|  | Broilers | | | | |  | Turkeys | | | | |
| ***Salmonella*** | 2013 | 2014 | 2015 | 2016 | 2017 |  | 2013 | 2014 | 2015 | 2016 | 2017 |
|  | **68** | **74** | **72** | **73** | **65** |  | **39** | **27** | **47** | **50** | **47** |
| 0 Class/Susceptible | 79% | 74% | 53% | 51% | 55% |  | 56% | 19% | 49% | 4% | 19% |
| 1 Class | 4% | 8% | 0% | 5% | 6% |  | 8% | 19% | 4% | 6% | 21% |
| 2–3 Classes | 10% | 18% | 13% | 44% | 38% |  | 31% | 59% | 38% | 90% | 57% |
| 4–5 Classes | 6% | 0% | 35% | 0% | 0% |  | 5% | 4% | 9% | 0% | 2% |
| 6–7 Classes | 0% | 0% | 0% | 0% | 0% |  | 0% | 0% | 0% | 0% | 0% |
|  | 100% | 100% | 100% | 100% | 100% |  | 100% | 100% | 100% | 100% | 100% |
|  | Broilers | | | | |  | Turkeys | | | | |
|  | **2013** | **2014** | **2015** | **2016** | **2017** |  | **2013** | **2014** | **2015** | **2016** | **2017** |
| ***Campylobacter*** | **27** | **26** | **25** | **31** | **44** |  | **87** | **85** | **106** | **79** | **80** |
| 0 Class/Susceptible | 41% | 35% | 32% | 65% | 52% |  | 62% | 39% | 34% | 47% | 29% |
| 1 Class | 19% | 38% | 44% | 23% | 20% |  | 24% | 39% | 57% | 42% | 34% |
| 2–3 Classes | 41% | 27% | 24% | 13% | 27% |  | 14% | 22% | 9% | 11% | 38% |
| 4–5 Classes | 0% | 0% | 0% | 0% | 0% |  | 0% | 0% | 0% | 0% | 0% |
| 6–7 Classes | 0% | 0% | 0% | 0% | 0% |  | 0% | 0% | 0% | 0% | 0% |
|  | 100% | 100% | 100% | 100% | 100% |  | 100% | 100% | 100% | 100% | 100% |

*Temporal changes in multiclass resistance prevalence are expressed as percent change in multiclass resistance (i.e., current year or initial surveillance year minus previous year divided by the previous year or initial surveillance year then multiplied by 100).*
